# A Talk between Flavonoids and Hormones to Reorient the Growth of Gymnosperms

**DOI:** 10.3390/ijms222312630

**Published:** 2021-11-23

**Authors:** Luis Morales-Quintana, Patricio Ramos

**Affiliations:** 1 Multidisciplinary Agroindustry Research Laboratory, Instituto de Ciencias Biomédicas, Facultad de Ciencias de la Salud, Universidad Autónoma de Chile, Talca 3467987, Chile; 2 Centro de Investigación de Estudios Avanzados del Maule (CIEAM), Vicerrectoría de Investigación y Postgrado, Universidad Católica del Maule, Talca 3460000, Chile; 3 Centro de Biotecnología de los Recursos Naturales (CenBio), Facultad de Ciencias Agrarias y Forestales, Universidad Católica del Maule, Talca 3460000, Chile; 4 Centro del Secano, Facultad de Ciencias Agrarias y Forestales, Universidad Católica del Maule, Talca 3460000, Chile

**Keywords:** conifers, hormone and flavonoids distribution, inclination response, lignin biosynthesis

## Abstract

Plants reorient the growth of affected organs in response to the loss of gravity vector. In trees, this phenomenon has received special attention due to its importance for the forestry industry of conifer species. Sustainable management is a key factor in improving wood quality. It is of paramount importance to understand the molecular and genetic mechanisms underlying wood formation, together with the hormonal and environmental factors that affect wood formation and quality. Hormones are related to the modulation of vertical growth rectification. Many studies have resulted in a model that proposes differential growth in the stem due to unequal auxin and jasmonate allocation. Furthermore, many studies have suggested that in auxin distribution, flavonoids act as molecular controllers. It is well known that flavonoids affect auxin flux, and this is a new area of study to understand the intracellular concentrations and how these compounds can control the gravitropic response. In this review, we focused on different molecular aspects related to the hormonal role in flavonoid homeostasis and what has been done in conifer trees to identify molecular players that could take part during the gravitropic response and reduce low-quality wood formation.

## 1. Introduction

Trees represent the most important source of biomass production, the major sink for atmospheric CO_2_ overloads, and an environmentally friendly renewable alternative to fossil fuel on the planet Earth. Population growth will increase the world demand for wood for buildings and pulp for paper in the near future [1]. In the setting of global change and with the goal of improving wood quality, sustainable management of forests is vital. To solve these problems, silvicultural practices will need to be optimized, and the application of modern genomic techniques should be useful to quickly improve the genetic material used in industrial plantations [2,3,4,5]. This smart breeding considers the use of genomics to identify key molecular players involved in wood formation through new translational genomic approaches such as marker-assisted selection or genomic selection, which should improve its efficiency by providing relevant early selection criteria supported by DNA markers. In addition to the exploitation of the variation observed in nature, genetic engineering is an efficient alternative to support the domestication of trees, which requires the identification of target genes to be modified. For this reason, it is important to understand the molecular, genetic, hormonal, environmental, and ontogenic factors involved in wood formation, taking into consideration that these factors control the formation of different types of wood observed in the same tree, showing different chemical, mechanical and physical properties that affect wood quality.

In response to environmental factors, which can affect normal growth and development, plants modify their growth patterns. This ability is fundamental for plant survival. The biochemical mechanisms by which plants perceive light and gravity stimuli are not fully clear. However, numerous studies have reported that their perception initiates a signal transduction cascade, leading to a response through differential growth [6,7,8,9]. In the case of trees, reorientation to the normal gravity vector growth involves not only wood formation but also a primary gravitropic reaction where plants exert a physical force to restore upright growth [10].

This article reviews the current knowledge about molecular and biochemical aspects involved in the relationship between secondary metabolites such as flavonoids and lignin, and hormones in the recovery of vertical growth and the consequent low-quality wood formation in gymnosperm tree species.

## 2. Gravitropism in Gymnosperms

In herbaceous plants, the response to gravitropic stimuli depends on the differential elongation of the affected organ, which means that the growth rate on the lower side of inclined stems is higher than that on the upper side, restoring upward growth. In the case of trees, the reorientation of stems and branches with secondary tissues must be brought about by some mechanism involving bending since, lignified woody stem cells cannot be elongated. To this end, branches and stems of trees exposed to gravitropic stimuli display asymmetric radial growth, which generates “reaction wood” [11]. This kind of wood is called compression wood (CW) in gymnosperms, and it is formed on the lower side of tilted stems of gymnosperms due to gravitropic stress [11]. 

Stem reorientation involves wood formation as part of a gravitropic reaction where tissues display a physical strain while trying to reorient to a vertical position [12]. This response is unilateral and creates physical wood strains that force the stem back toward its original vertical orientation and it shows morphological and molecular differences in gymnosperms compared to angiosperm species [13,14]. 

Specifically, in gymnosperms or conifer species, several studies have reported genes and proteins that can be regulated in response to inclination or a loss of verticality [15,16,17,18,19,20,21,22,23]. Based on this information it has been noted that plant hormones seem to be important signaling molecules mediating this response (see Table 1).

## 3. Hormones and the Gravitropic Response in Gymnosperms

Numerous studies have reported an association between the gravitropic response and hormones such as auxins, ethylene, and cytokinin, and their distribution along the organs affected [6,7,32,33,34]. In this regard, the phytohormone distribution affects the cellular processes involved in cell division, cell expansion, and cellular differentiation processes, influencing the development of different types of wood related to the response to gravitropic stimuli of trees [35,36,37].

Auxin is a well-known hormone related to leaf vascular tissue and tracheid development [27]. In *Pinus radiata* d. Don (radiata pine) seedlings, studies suggest that IAA is distributed differentially in the stem after inclination stimuli [32]. The authors evaluated, through microscopy using an immunodetection approach, the differential distribution of IAA with antibodies against auxin phytohormone in sections of young seedlings of radiata pine exposed to tilting, detecting a higher concentration of auxin on tracheids that showed typical morphological characteristics of CW. Recently, the auxin content was quantified at different times of inclination of young seedlings of radiata pine [23]. In this work, the authors showed a time course progression of the auxin concentration along the inclined radiata pine seedlings. Both experimental approaches agreed with the fact that auxin is differentially distributed, and this is further supported by the expression analysis of auxin-targeted genes. The transcriptional profile analysis of an auxin-repressed protein gene (ARP) showed downregulation by auxin, which was demonstrated by treatments with naphthalene-acetic acid (NAA) and reduced expression on the lower side of the inclined seedlings.

Ethylene (C_2_H_4_) is a gaseous phytohormone involved in many aspects of plant growth and development, such as seed germination, fruit ripening, and responses to biotic and abiotic stresses [38,39,40], and it is involved in determining certain aspects of the tree form [41]. In *Pinus sylvestris*, high ethylene production is concomitant with the seasonal period of wood development compared to dormant trees [28]. Ethylene is produced in CW [25], and the role of endogenous ethylene in wood forming tissues at the molecular level has been described [26,27,42]. For instance, leaning treatments induced not only CW but also increased ethylene biosynthesis on the CW-developing half of the tilted stem [26]. Similarly, ethylene precursor 1-aminocyclopropane-1-carboxylate (ACC) was detected in cambium from the CW- side but not on the opposite side of the *Pinus contorta* branches [24]. 

Gene expression analysis has been performed in conifers such as *Pinus taeda* [29] and *Pinus pinaster* [17,35]. In radiata pine, a transcriptional analysis was conducted to evaluate the expression of key genes involved in the ethylene biosynthesis pathway, showing that 1-aminocyclopropane-1-carboxylate synthase (ACS) and 1-aminocyclopropane-1-carboxylate oxidase (ACO) genes are upregulated in inclined stems [30]. A study of treatments with exogenous ethylene application to radiata pine seedlings showed that the morphological characteristics of CW and lignin deposition on cell walls of the stem of inclined seedlings were accelerated compared to nonethylene-treated seedlings [31]. 

Jasmonic acid (JA) is a phytohormone ubiquitous in the plant kingdom [43] that regulates biological processes and secondary metabolite pathways in plants, including flavonoid biosynthesis [43,44]. Active jasmonate (JA-Ile) can induce the degradation of transcriptional repressors called the jasmonate ZIM-domain (JAZ) mediated by the SCF-COI1 complex, thus releasing the transcription factor MYC2 to play a regulatory role in gene expression [45]. 

Recently, a relationship was reported between IAA and JA in radiata pine. Thus, JA displays higher accumulation in the upper part, while IAA was found to be higher in the opposite half of the JA distribution of inclined stems [23]. This would partially explain the differences in the molecular program activated on both sides of the inclined stem [20]. Salazar et al. [23] observed that a higher lignin content was deposited in the lower half of the stem after extended times of inclination, with H-lignin being the most accumulated monomer in the lower half and G-lignin units predominating in the upper half (see Table 2). This differential composition of lignin on both sides of the stem could be under hormonal regulation, mainly modulated by the content of IAA and JA [23].

Analyses were performed in stems from inclined seedlings divided into upper and lower halves. Different letters denote significant differences between both sides of the stem (*p* < 0.05; ANOVA) (Table adapted from Salazar et al. [23]).

## 4. Hormone and Flavonoid Metabolism during the Gravitropic Response

Auxin and ethylene are phytohormones implicated in a wide range of biological processes, including elongation, lateral root formation, and gravitropic reactions in roots [46,47,48]. Auxin needs to move from its synthesis site to different parts where exerts its function [49]. Auxin transporters, which include a large number of ABCB, AUX1/LAX, and PIN proteins, modulate auxin fluxes within plants [49,50,51].

Since the role of auxins in the gravitropic response was first suggested by Went [52], substantial progress in knowledge about the signal transduction involved in this biological process has been reported. Indeed, an asymmetrical distribution of IAA was observed in response to gravitropic stimulation of the coleoptile of maize and rice [53,54]. Furthermore, the molecular mechanism underlying asymmetric auxin distribution during the gravitropic response is not fully clear. To date, three main mechanisms have been suggested to explain this process. One proposes that the transduction mechanism involves targeting the auxin efflux facilitator protein PIN3 to the plasma membrane in response to gravitropic stimulation [55]. The second mechanism comprises phosphorylation of proteins in a reversible manner, which may modulate the activity, abundance, or localization of auxin transport proteins [56,57,58]. A third mechanism suggests that the response to gravity is regulated by localized synthesis or directed transport of small molecules that regulate auxin transport [59,60,61,62]. In the latter proposed mechanism, these small molecules are flavonoids. These molecules play paramount roles in essential biological processes of plants, including auxin transport regulation [63]. Flavonols are aromatic compounds within the flavonoid family, and several environmental cues modulate their biosynthesis [64]. One of the molecular functions in some specific tissues may be associated with auxin transport modulation and consequently, can affect auxin-related biological processes, including gravitropism and branching [61,65,66]. ABC transporters belonging to the ABCB/p-glycoprotein/multidrug resistance-like MDR/PGP family act as ATP-dependent auxin transporters, and their interaction with PIN proteins influences the directionality and substrate specificity of the auxin efflux machinery [67,68]. In *Arabidopsis*, studies suggest that quercetin inhibits the auxin efflux activity of the MDR/PGP transporter [67,69] Moreover, in *Arabidopsis* auxin transport is affected by flavonol glycosides [70,71], specifically inducing changes in PIN2 polarity mediated through a phosphorylation mechanism [72].

Researchers have shown that auxin MDR/PGP transporters are directly [68,69,72] or indirectly [73] (Bailly et al., 2008) regulated by flavonols. Additionally, studies of mutant *Arabidopsis* plants without flavonoid biosynthesis showed elevated auxin transport in several tissues due to mutations of chalcone synthase (CHS) encoding gene [59,60]. This result is consistent with the effects observed in basipetal auxin transport in a CHS-silenced *Arabidopsis* mutant [74]. Reports have suggested that flavonoids promote gravitropism in roots presumably by reducing auxin transport to the root tip by inhibiting PIN-MDR/PGP complex activity, which modulates the differential growth [61]. In contrast, Pourcel et al. [75] showed that in *Arabidopsis* with a mutated chalcone isomerase (CHI) gene, the lack of flavonoid production and chemical rescue assays did not affect the expression of auxin-responsive genes.

Like several secondary metabolites, flavonoids are synthesized within the cytosol and stored within the vacuole. The mechanisms involved in the transport of flavonoids into the vacuole are not yet fully clear. There are two proposed main transport mechanisms: the first comprises membrane vesicle– and membrane transporter-mediated transport [76,77]. The second mechanism proposes the participation of ATP binding cassette (ABC) proteins and multidrug and toxic extrusion (MATE) in anthocyanin transport [77,78,79,80]. This model proposes that vacuolar transport of flavonoids occurs through directly energized ABC-type transporters [80,81,82] or secondarily energized MATE antiporters, usually driven by the H+ gradient across the tonoplast [78,83]. The participation of MATE was initially determined in *Arabidopsis* through the characterization of the MATE transporter Transparent Testa 12 (TT12), which acts as a flavonoid/H+- antiporter on the vacuolar membrane and has substrate-specific activity [77,78,79]. TT12 is recognized by the transport capacity of anthocyanins and flavan-3-ols glycosylated [78]. This means a possible positive regulation of flavonol levels that induces the expression level of biosynthetic genes of flavonoids, providing feedback. In *Medicago truncatula*, two MATE transporters were identified by observing vacuolar subcellular localization, and their action on flavonoid sequestration was established [77,79]. Several lines of evidence suggest the involvement of ABC transporters, particularly from the ABCC subfamily -also called multidrug resistance proteins (MRPs)- in vacuolar flavonoid sequestration [80,82,84]. In this regard, a study in *Zea mays* showed that MRP3, an ABCC-type transporter, delivers anthocyanins to the vacuole [84]. In grapevine, a complete genome analysis identified a gene, that encodes a tonoplast-localized ABCC1 protein able to transport anthocyanin 3-glucosides into the vacuole [82]. Interestingly, ABC and MATE, which are involved in flavonoid transport, are upregulated in *Arabidopsis tt5* mutants supplemented with naringenin, indicating that flavonoid accumulation induction must be stored or redistributed [75]. Recently, studies of *Arabidopsis* showed that an ABC transporter, specifically AtABCC2, can transport anthocyanins, flavones, and flavonol glycosides [80].

Concerning to ethylene and flavonoid relationship, experiments in *Arabidopsis* roots suggested that ethylene modulate the accumulation of flavonoids, affecting the root auxin transport, gravitropism, and elongation growth [85]. In addition, ethylene and auxin regulate the balance of flavonol biosynthesis in which quercetin is the active modulator of auxin transport that regulates the growth processes, such as root gravitropism [86]. Despite this, no information is available about the relation between ethylene and flavonoid biosynthesis in gravitropic response and wood development.

The understanding of the subcellular localization, transport, and storage of flavonoids in plants, particularly in trees, remains unclear [87]. Regarding flavonoid homeostasis during the gravitropic response in wood-forming tissues of pine (see Table 3), putative transporters with high sequence identity to MATE (AtTT12) and ABCC (MRPs) were identified in SSH libraries [21]. These transporters were characterized in their expression during inclination stress and in response to hormones, showing robust auxin signaling dependence [88,89].

Additionally, the expression pattern of genes associated with the phenylpropanoid biosynthetic pathway was studied. One study was performed to correlate the hormonal imbalance because of flavonoid differential accumulation with consequences for secondary cell wall modifications [32]. In this study, the authors reported an upregulation of genes involved in the flavonoid biosynthetic pathway as chalcone synthase (CHS) and flavanone 3-hydroxylase (F3H), and a specific gene encoding for flavonol synthase (FLS) on the upper side of the tilted stem, while on the opposite side, those genes were strongly repressed. Concomitantly, a target gene of auxin signaling (ARP) in addition to genes related to monolignol biosynthesis, such as cinnamoyl-CoA reductase (CCR) and caffeic acid *O*-methyltransferase (COMT), were upregulated on the inferior side of the tilted stem [32].

Consistent with these findings, the flavonols quercetin and kaempferol accumulated in the upper half, and a high auxin content was detected on the opposite side of the inclined stem [32]. Flavonoid biosynthesis is controlled by the combined action of transcription factors (TFs) and it is expressed in a very spatially and temporally specific manner [90,91]. Recently, the identification and transcriptional characterization of MYB TFs were assessed in radiata pine exposed to inclination stress [88]. Additionally, the promoter region of an ABCC transporter (PrMRP1) was isolated, and it contained putative cis-regulatory elements for MYBs and auxin-responsive elements [88].

**Figure 1:**
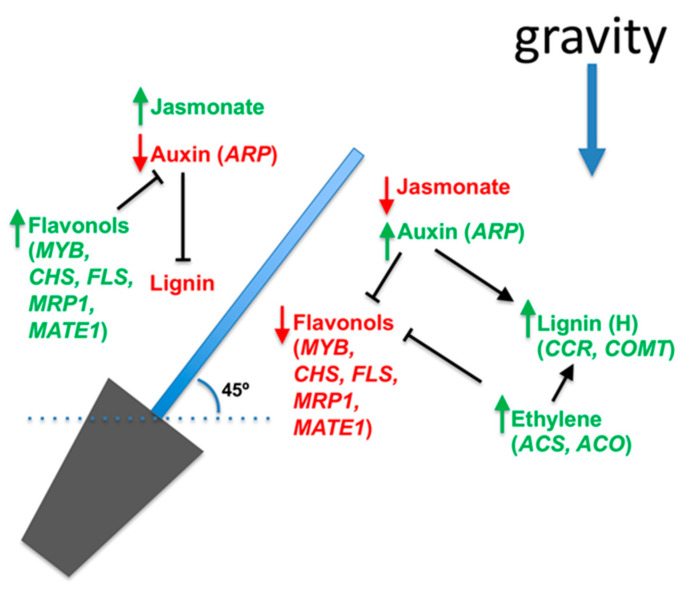
Summary of hormonal distribution, genetic expression profile, and metabolic differential accumulation in gymnosperms exposed to inclination. Auxin-Repressed Protein (ARP), chalcone synthase (CHS), flavonol synthase (FLS), cinnamoyl-CoA reductase (CCR), caffeic acid O-methyltransferase (COMT), MYB transcription factors, flavonoid transporters as ABCC-flavonoid transporter (multidrug transporter protein 1 (MTR1) and multidrug and toxic extrusion 1 (MATE1) are genes differentially expressed on both sides of the inclined stem of radiata pine. Auxin content, ethylene as wells as ethylene biosynthesis genes (ACC synthase [ACS] and ACC oxidase [ACO]), and jasmonate content are differentially distributed on both sides of the inclined stems and regulate the differential expression of genes involved in lignin and flavonol biosynthesis (based on Salazar et al. (2020) [23], Ramos et al. (2016) [32], Klintborg et al. (2002) [28], Little and Eklund (1999) [26], Ramos et al. (2012) [30], Ramos and Herrera (2013) [31], Gomez et al. (2018) [88], Morales-Quintana et al. (2019) [89]).

Finally, we summarized the current knowledge about the hormonal distribution, the differential transcriptional profiles of genes involved in flavonol and monolignol biosynthetic pathways, and the differential accumulation of their specific secondary metabolites. In radiata pine seedling, at the lower half of inclined stem, auxin, and ethylene increase [23,30], which is concomitant with a higher lignin accumulation [23,31]. These observations correlate with a modulation in the expression of target genes of auxin (*ARP*) [32], and also with biosynthetic genes of lignin, ethylene, and biosynthesis and transport of flavonols. Auxin regulates negatively the genes involved in the flavonol biosynthesis, and positively with the genes involved in the lignin biosynthesis, and the same effect was reported in plants treated with Ethrel [31]. On the opposite half of the inclined stem (upper half), auxin displays a decrease in their accumulation and distribution, meanwhile jasmonate increases. The low content of auxin releases the repression of genes related to flavonols biosynthesis allowing to increase their content that blocks the auxin distribution and, in consequence, auxin does not induce the genes related to lignin biosynthesis [23]. Jasmonate acts contrary to the effects of auxin hormone, which is accumulated in the upper half and decrease in the lower half of inclined stems [23] (Figure 1).

## 5. Conclusions

Tree reorientation to natural gravity vector growth involves a primary gravitropic reaction where plants exert a physical force intending to restore upright growth, which results in abnormal wood formation. Thus, it is necessary to investigate the time course of the differential accumulation of hormones and the secondary metabolites in trees exposed to tilting stress. Despite the vast knowledge, all of the information to date has been mainly obtained from model plants, which are principally angiosperms, and very few have been validated in trees and even fewer in gymnosperms. Here, we present the collected evidence about genetic, metabolic, and hormonal participation in the response to inclination in a conifer species. This is an initial approach to describe and understand the relationship between flavonoids and hormones that could underlie the molecular response to recover vertical growth in conifer trees without affecting wood quality. Identification of the key molecular players that can be regulating the hormone distribution, which based on the presented evidence, could be helpful to design new strategies to obtain trees with a less prone to develop compression wood and, in consequence, less wood with low quality (higher lignin and lower cellulose content).

Finally, to better understand the response to tilting stress in conifer trees, genomic and transcriptomic approaches, among other omics approaches, will be helpful. Promoter sequence identification and gene isolation to characterize and develop new strategies to improve wood quality and timber production are also key challenges. All of these experimental approaches are of paramount importance to face the future challenge of global demand from climate change and population growth.

## Figures and Tables

**Table 1:** Summary of the principal researches about proteins families, biological processes, hormones signaling and biosynthesis, modulated in response to inclination in gymnosperm species.

Species	Genes/Proteins Families and Biological Processes	Reference
*Pinus contorta*	Ethylene biosynthesis—ACC accumulation	[24]
*Picea abies*	Ethylene biosynthesis—ethylene induce changes in cell walls composition	[25]
*Pinus taeda* L.	Lignin biosynthesis—Phenylalanine amonio lyase (PAL), Cinnamate-4-hydroxylase (C4H), O-methyltransferase (OMT), 4-Coumarate-CoA ligase (4CL), and Cinnamyl alcohol dehydrogenase (CAD). Cell wall carbohydrate metabolism—Xyloglucan endotransglycosylases (XET). Transcription factors—MADS box, homeodomain, LIM-domain proteins	[15]
*Abies balsamea*	Ethylene biosyntesis—differential ethylene accumulation in tilted seedlings and tracheid production	[26]
*Pinus taeda* L.	Arabinogalactan proteins (AGPs)—differential accumulation of secondary cell walls remodelling proteins	[16]
*Pinus pinaster* Ait.	Ethylene and lignin biosynthesis—ACC oxidase, caffeic O-methyltransferase and caffeoyl CoA-O-methyltransferase. Nitrogen and carbon assimilation—glutamine synthetase and fructokinase.	[27]
*Pinus sylvestris*	Ethylene biosynthesis—ACC synthase and ACC oxidase activity	[28]
*Pinus pinaster* Ait.	Cell wall formation—glycine-rich protein (GRP) and UDP-glucose pyrophosphorylase.	[17]
*Pinus taeda* L.	Cell wall-related proteins—cellulose synthase, expansin, xyloglucan endotransglycosylases (XET), glucanase, laccase, arabinogalactan-proteins (AGPs). Intermediate metabolism—12-OXO-phytodiennoate reductase, UDP-glucosyltransferase, Short-chain type dehydrogenase/reductase, Myo-inositol-1-phosphate synthase, UDP-glucose pyrophosphorylase.	[29]
*Pinus pinaster* Ait.	Defense, carbohydrates and amino acid metabolisms, genes and proteins expression, cytoskeleton, cell wall biosynthesis, secondary and primary metabolisms.	[18]
*Chamaecyparis obtusa*	Cell wall modification proteins—β-1,3-glucanase-like protein. Lignin biosynthesis—laccase.	[19,20]
*Pinus radiata* D. Don	Hormone signaling—EIN3-like protein, Auxin-repressed protein. Phenylpropanoid pathway—Phenylalanine amonio lyase (PAL), chalcone synthase (CHS), flavanone 3-hydroxylase (F3H).	[21]
*Pinus radiata* D. Don	Ethylene biosynthesis—ACC oxidase and ACC synthase differetially expressed.	[30]
*Pinus radiata* D. Don	Cell division, cellulose biosynthesis, lignin deposition, microtubules.	[22]
*Pinus radiata* D. Don	Ethylene signaling—Induction of tracheids with compression wood phenotypes in seedlings treated with ethylene biosynthesis precursor.	[31]
*Pinus radiata* D. Don	Lignin and flavonoid biosynthesis—Chalcone synthase (CHS), Flavanone 3-hydroxylase (F3H), Flavonol synthase (FLS), Caffeic acid O-methyl transferase (COMT), Cinnamoyl-CoA reductase (CCR), auxin signaling—Auxin repressed-protein (ARP).	[32]
*Pinus radiata* D. Don	Auxin transporters—ABCB1, ABCB2, AUX1-1, AUX1-2, AUX1-3 and PIN1, lignin biosynthesis—analysis of lignin content and monomeric composition. Auxin and jasmonate content and distribution.	[23]

**Table 2:** Lignin content and monomeric composition in seedlings of radiata pine exposed to 1 month of tilting.

	1 Month	
	Stem lower half	Stem upper half
KL (mg/gAIR)	417.5 ± 18.4 ^a^	367.4 ± 5.7 ^b^
%H	47.9 ± 5.1 ^a^	16.9 ± 0.3 ^b^
%G	52.1 ± 5.1 ^b^	83.1 ± 0.3 ^a^
G/H	1.1	4.9

Different letters indicate significant differences between samples (*p* < 0.05).

**Table 3:** Summary of genes and transporters involved in flavonoids homeostasis modulated in response to inclination in gymnosperm species.

Species	Genes/Proteins Involved in Biological Processes	Reference
*Pinus radiata* D. Don	*Phenylalanine amonio lyase* (*PAL*), *chalcone synthase* (*CHS*), *flavanone 3-hydroxylase* (*F3H*), *transparent testa 12* (*TT12*)—inclination response and flavonoids homeostasis	[21]
*Pinus radiata* D. Don	*Chalcone synthase* (*CHS*), *Flavanone 3-hydroxylase* (*F3H*), *Flavonol synthase* (*FLS*), *Caffeic acid O-methyl transferase* (*COMT*), *Cinnamoyl-CoA reductase*(*CCR*)—lignin and flavonols biosynthesis.	[32]
*Pinus radiata* D. Don	*ABCC-flavonoid transporter* (*MRP1*), *MYB2*, *MYB5*, *MYB6*, *MYB10*—flavonoid biosynthesis and homeostasis.	[88]
*Pinus radiata* D. Don	*MATE-flavonoid transporter* (*MATE1*)—intracellular flavonoid homeostasis	[89]
